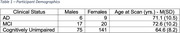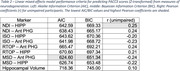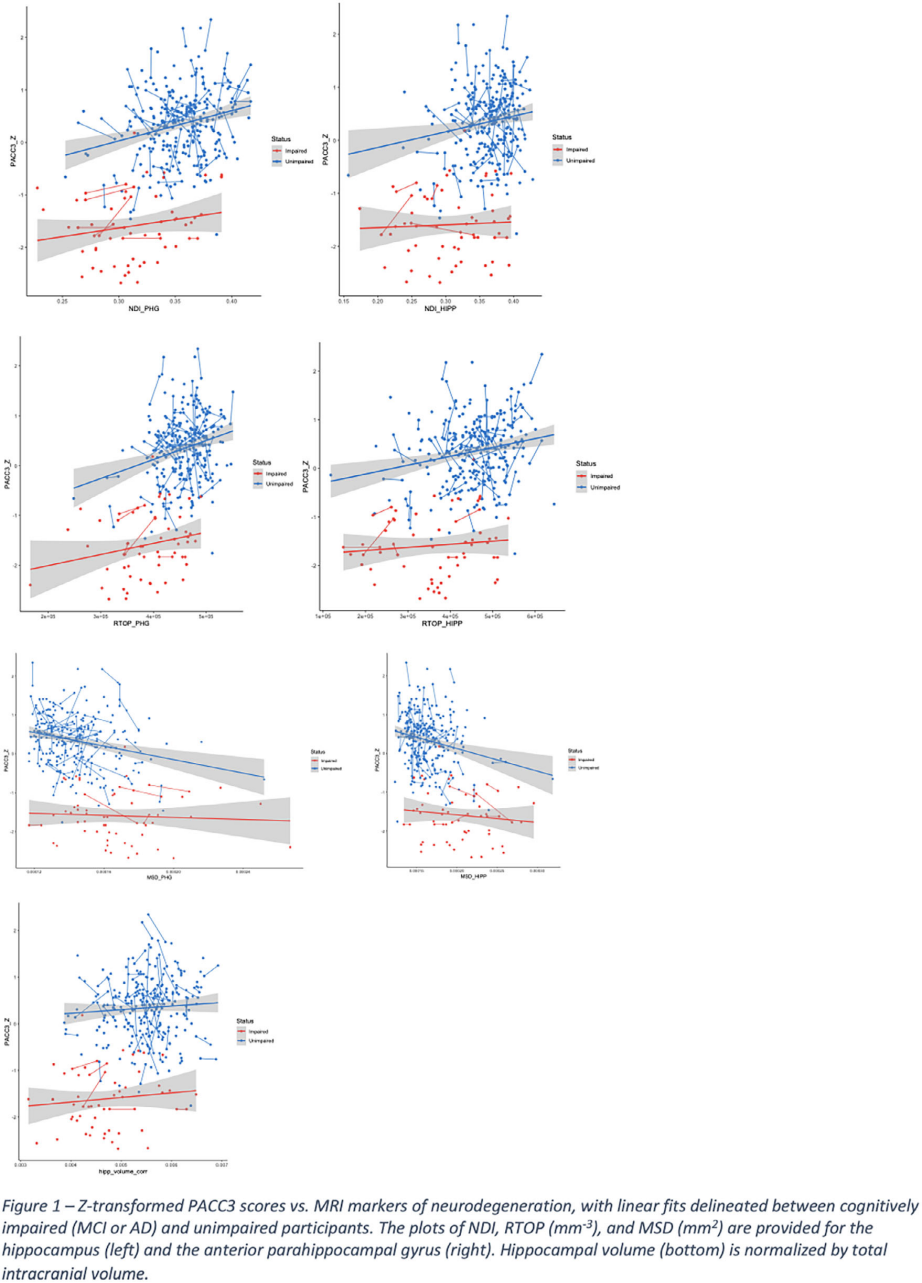# Neuroimaging Markers of Microstructural Neurodegeneration are More Sensitive to Cognitive Dysfunction than Hippocampal Volume in Early Alzheimer’s Disease: A Mixed‐Longitudinal Study

**DOI:** 10.1002/alz.091915

**Published:** 2025-01-09

**Authors:** Jason F Moody, Erin M. Jonaitis, Rebecca E. Langhough, Douglas C Dean, Andrew L Alexander, Sterling C. Johnson, Barbara B. Bendlin

**Affiliations:** ^1^ Wisconsin Alzheimer's Disease Research Center, University of Wisconsin‐Madison, School of Medicine and Public Health, Madison, WI USA; ^2^ Wisconsin Alzheimer’s Institute, University of Wisconsin School of Medicine and Public Health, Madison, WI USA; ^3^ Department of Pediatrics, University of Wisconsin‐Madison, Madison, WI USA; ^4^ Waisman Center, University of Wisconsin‐Madison, Madison, WI USA; ^5^ Department of Medical Physics, University of Wisconsin‐Madison, Madison, WI USA; ^6^ Department of Psychiatry, University of Wisconsin‐Madison, Madison, WI USA; ^7^ Geriatric Research Education and Clinical Center, William S. Middleton Memorial Veterans Hospital, Madison, WI USA; ^8^ Wisconsin Alzheimer's Disease Research Center, University of Wisconsin School of Medicine and Public Health, Madison, WI USA

## Abstract

**Background:**

While magnetic resonance imaging (MRI) markers of neurodegeneration are nonspecific to Alzheimer’s disease (AD) pathology, they have been correlated with cognitive dysfunction, and therefore, provide important information pertaining to disease staging. Neurodegeneration in AD is commonly assessed with macrostructural measures of brain atrophy, such as hippocampal volume. However, recent investigations have shown that markers of neural microstructure derived from diffusion MRI (DWI) may provide supplementary insight into the progression of AD pathophysiology. Furthermore, DWI measures of neurite density index (NDI), isotropic volume fraction (ISO), return to origin probability (RTOP), and mean squared displacement (MSD) have recently been associated with cerebrospinal fluid markers of senile plaques, neurofibrillary tangles, and AD‐related neurodegeneration.

In this study, we used linear mixed‐effects (LME) modeling to compare the utility of different MRI markers for predicting performance on a three‐test Preclinical Alzheimer’s Cognitive Composite (PACC3) in 268 late‐middle‐aged participants.

**Methods:**

268 participants from the Wisconsin Registry for Alzheimer’s Prevention and the Wisconsin Alzheimer’s Disease Research Center (216 unimpaired, 37 MCI, 15 AD) were imaged between 1 and 2 times with T1‐weighted MRI and multi‐shell DWI (Table 1). For each scan, hippocampal volume was estimated and average NDI, ISO, RTOP, and MSD values were extracted from the hippocampus and anterior parahippocampal gyrus. PACC3 scores were calculated from comprehensive neuropsychological testing within 6 months of imaging. Neuroimaging metrics were used as predictors in separate LME models of the following form:

PACC3 = b_0_ + b_1_*(metric) + b_2_ *(age) + b_3_*(sex) + b_4_*(metric*clinical status) + b_5_*(years of education) + u_i_

**Results:**

DWI metrics consistently outperformed hippocampal volume in predicting PACC3 scores across both brain regions (Table 2). Anterior parahippocampal MSD exhibited the lowest AIC/BIC values, while hippocampal NDI had the strongest association with PACC3 scores among unimpaired participants. Meanwhile, hippocampal volume had the largest AIC/BIC values and the weakest correlation with PACC3 scores (Table 2, Figure 1).

**Conclusion:**

Our study provides evidence that neuroimaging markers of brain microstructure may be particularly sensitive to subtle alterations predictive of cognitive performance, especially early in AD. Future work will translate these analyses to larger cohorts and incorporate fluid markers of amyloid and tau.